# Biomass allocation and plant morphology explain the difference in shrub species abundance in a temperate forest

**DOI:** 10.1002/ece3.10774

**Published:** 2023-12-03

**Authors:** Rudiger Markgraf, Frédérik Doyon, Sylvain Delagrange, Daniel Kneeshaw

**Affiliations:** ^1^ Département des sciences biologiques Université du Québec à Montréal, UQAM Montréal Quebec Canada; ^2^ Département des Sciences Naturelles Université du Québec en Outaouais, UQO Gatineau Quebec Canada; ^3^ Institut des Sciences de la Forêt Tempérée, ISFORT Ripon Quebec Canada

**Keywords:** biomass allocation, functional traits, gap‐forest environment, leaf traits, ontogeny, persistence, plant morphology, shrub strategies

## Abstract

In forested ecosystems, shrubs must succeed in persisting in low‐light environments, while simultaneously having the ability to rapidly expand and occupy newly created canopy openings, yet little is known about the traits that make this possible. We hypothesize that shrub species that are abundant in the understory exhibit a specific set of functional traits that define their ability to persist during unfavorable periods and to rapidly exploit newly created habitats. We tested this by comparing field‐measured functional traits such as biomass allocation, leaf display, crown morphology, and leaf traits, across individual size classes and two gap‐forest environments of five shrub species. We observed significant differences in traits between species, size classes, and gap‐forest environments. These differences were primarily related to biomass allocation traits, followed by leaf display, crown morphology, and leaf traits. Abundant shrubs like mountain maple (*Acer spicatum*) and hazelnut (*Corylus cornuta*) invested significantly more biomass in roots, had a larger total leaf area, and displayed leaves in a more efficient manner to intercept light. The high investment in root biomass can be interpreted as shrubs exploiting the persistence and colonization strategy through resprouting. Permanent sub‐canopy status likely explains the importance of efficient leaf display, wherein abundant shrubs had a large leaf area with minimal support structures.

## Graphical abstract

We found that abundant shrub species display a specific set of traits that are adapted to the forest understory. We observed abundant shrub species invest in root biomass allocation, leaf display, and monolayer crowns, as opposed to less abundant shrubs.

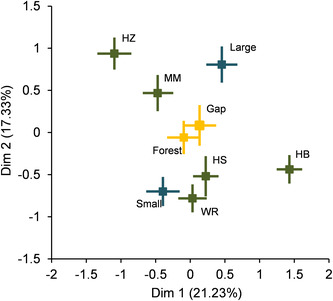

## 1 INTRODUCTION

With the increase in intermediate disturbances in forests (sensu Woods, 2000), the buildup of dense shrub understory layers that inhibit tree regeneration establishment and growth has been observed in different parts of the world (Royo & Carson, 2006). However, understory shrubs do not have a strategy of height growth and dominance similar to trees, instead in forested systems, they are mostly constrained to understorey environments. Successful adaptations to ephemeral conditions require plant traits that allow abundant shrubs to quickly adjust carbon allocation for persistence in low‐light environments, in addition to fast vegetative growth for rapid colonization of newly created canopy openings (Aubin et al., 2005, 2016; Bell et al., 2011; Bond & Midgley, 2001, 2003; Fraterrigo, 2016; Young & Peffer, 2010).

However, bio‐physical constraints limit the range of traits that will be expressed due to life‐history trait trade‐offs (Wright et al., 2010). For example, shade‐intolerant species allocate more biomass to the stem to access the full light of the canopy (Kitajima, 1994; Messier et al., 1999), while shade‐tolerant species allocate more to the root system (Delagrange et al., 2004; Gaucher et al., 2005; Gleeson & Tilman, 1994). Yet despite research on these trade‐offs in trees, for example, shade‐intolerant trees invest in height growth and high branching to maximize light capture (Claveau et al., 2002; Delagrange et al., 2004; Messier et al., 1999; Niinemets, 2010), while shade‐tolerant trees invest in wide monolayer crowns for light capture and survival in understorey environments (Messier et al., 1999), how trade‐offs play out in shrubs is not well known. Directly transferring knowledge on trees to shrubs is hazardous as tree species have an architectural legacy constrained by investment in stem biomass, whereas shrub species have been observed to rapidly reshape their morphology as a function of resource availability through height reduction and branch shedding (Aubin et al., 2005).

According to the leaf economics spectrum, species with higher SLA (specific leaf area) typically should have higher photosynthetic capacity, higher dark respiration, higher leaf N concentration, shorter leaf longevity, and lower defense against herbivory (Reich et al., 1999, 2003; Wright et al., 2004) and thus faster growth. We would thus expect this of the abundant shrubs in large gaps, whereas species growing in the understory would have a longer leaf life span as it has been associated with longer nutrient retention and thicker leaf litter (Niinemets, 2006; Poorter, 2002; Westoby et al., 2002). The functional equilibrium hypothesis of biomass allocation (also known as the optimal partitioning theory) proposes that in response to stress, plants increase allocation for uptake of the most limiting resource (Freschet et al., 2015, 2018; Markesteijn & Poorter, 2009; Poorter et al., 2012). Allocation to roots should increase following a decrease in belowground resources (nutrients, water), and allocation to leaf surface area and stems increases following a decrease in aboveground resources (light) (McCarthy & Enquist, 2007; Poorter, 1999, 2002). Moreover, trends extend to plant morphology as increased light levels should result in a larger total leaf area (TLA), higher leaf area index (LAI), higher leaf display index (LDI), higher Crown H/D, and a smaller canopy area (Beaudet & Messier, 1998; Poorter, 1999; Poorter & Werger, 1999).

Furthermore, trait trade‐offs are also constrained by plant size. Ontogeny heavily impacts biomass allocation, crown morphology, architectural patterns, and leaf traits (Taugourdeau et al., 2019). Both stem mass fraction (SMF) and branch mass fraction (BMF) generally increase with increasing plant size, while allocation to root mass fraction (RMF) decreases (Delagrange et al., 2004; Poorter, 1999). Variation in crown morphology with ontogeny has been observed, for example, juvenile shade‐tolerant species exhibit flatter crowns to better intercept limited understory light (Messier et al., 1999), while adults receiving higher illumination would display a larger crown depth, a larger crown area and more leaf layers (Niinemets, 2010; Poorter, 1999; Poorter & Werger, 1999). Consequently, crown light interception efficiency is expected to decrease with ontogeny due to an increase in leaf overlap (Delagrange et al., 2006).

Therefore, to survive in the understory shrubs need to be plastic to adjust their traits to effectively use a “sit and wait” strategy under a shifting mosaic of suitable habitats (Aubin et al., 2005). Phenotypic plasticity in different environments and life stages should be characteristic of plants in environments showing high spatio‐temporal heterogeneity, (Alpert & Simms, 2002; Kumordzi et al., 2019; Pugnaire & Valladares, 2007).

What combination of traits allows shrub species to increase in abundance in disturbed landscapes and how do these change with the size of individuals and light environments? The goal of this study was to compare the functional traits related to biomass allocation, morphology, and leaves, between shrub species according to their abundance in a forest landscape perturbed by intermediate disturbances. By doing so, we aim to describe the traits associated with shrub species in changing forest environments (gaps and understory). We hypothesize that abundant shrub species will (1) exhibit gap specialist traits allowing fast exploitation of newly created suitable habitats (greater density and growth in gaps), (2) be characterized by an explicit set of persistence traits for surviving periods of low resources (high RMF), and (3) abundant shrubs will show more intraspecific variance in the expression of their functional traits in order to use the “sit and wait” strategy.

## 2 MATERIALS AND METHODS

### 2.1 Study site

Our study sites are located in the La Verendrye wildlife reserve, in the balsam fir/yellow birch bioclimatic domain of Quebec (Robitaille & Saucier, 1998). The average elevation is 389 m, and the most common surface deposit is a thin layer of till and bedrock material made of metamorphic rocks (Robitaille & Saucier, 1998). The mean annual precipitation in Maniwaki (the closest weather station) is 908.8 mm (including 238.3 mm as snow) and the mean annual temperature is 3.7°C. The mixedwood forests in these areas include balsam fir (*Abies balsamea*), yellow birch (*Betula alleghaniensis*), white spruce (*Picea glauca*), white birch (*Betula papyrifera*), black spruce (*Picea mariana*), white pine (*Pinus strobus*), eastern white cedar (*Thuja occidentalis*), trembling aspen (*Populus tremuloides*), red maple (*Acer rubrum*) and sugar maple (*Acer saccharum*). These southern mixedwood forests are naturally affected by predominantly small‐scale disturbances such as individual tree mortality, insect outbreaks, and windthrow (Prévost et al., 2003). The southern mixedwood yellow birch and balsam fir forest zone is heterogeneous due to the relatively short lifespan of balsam fir compared to the long‐life of yellow birch (Kneeshaw & Prévost, 2007). This heterogeneity was compounded by high grading diameter limit harvests (selection of large high‐quality stems) conducted from the 1960s to the 1980s and a spruce budworm outbreak (1980s), thereby resulting in an increased creation of canopy gaps and a higher level of intermediate disturbance (Doyon & Lafleur, 2004; Sabbagh et al., 2002).

### 2.2 Population‐level measures

To measure shrub density, we conducted sampling in 12 randomly selected 1 km^2^ landscapes. Within each landscape, we randomly sampled 18 sites. Nine sites were under the forest canopy and the nine other sites were three different sized gaps (small [40–200 m^2^], medium [201–600 m^2^], and large [601 m^2^+]), each replicated three times. Canopy gap size was measured assuming an elliptical shape (area = *π*ab). Irregular canopy gap shapes were avoided. Forest understory sites were sampled in circular plots (radius = 11.28 m, area = 400 m^2^). These four light conditions constituted the gap‐forest environment gradient. Shrub density measurements were made in 4 microquadrats of 5 m^2^ (radius of 1.26 m) in the forest understory sites and 4–8 microquadrats in the gaps, according to its size (small = 4, medium = 6, and large = 8). Microquadrats were distributed along northeast and northwest axes to account for variability in light conditions in the gap. We recorded shrub (height ≥ 20 cm, diameter at 10 cm < 1 cm) density by species in each microquadrat. For shrubs, basal sprouts and stem layering when detected were counted as one individual.

### 2.3 Trait sampling

Within four of the 12 landscapes, 32 sites were randomly sampled, 16 sites in the forest understory and 16 sites in 50–200 m^2^ gaps in order to evaluate the influence of environments with low and higher resource availability.

Direct and diffuse light were calculated using hemispherical photographs taken at the center of each site (for gaps where axes a and b of the ellipse meet). Photographs were taken at a height of 2 m, to capture the gap‐forest environment experienced by the shrubs. The camera was mounted onto a stand equipped with a leveling device to correctly identify the zenith and the camera was oriented so that the top of the photographs pointed toward geographic north (Beaudet & Messier, 2002). Diffuse and direct light were calculated using the Gap Light Analyzer software (GLA version 2.0).

Five shrub species were selected for this study: mountain maple, hazelnut, hobblebush (*Viburnum lantanoides*), honeysuckle (*Lonicera canadensis*) and wild raisin (*Viburnum cassinoides*). Sixteen traits were evaluated to explain shrub strategies (Table 1) (Cornelissen et al., 2003; Weiher et al., 1999). To control (or test) for ontogenic effect, two individuals, within each site, from each of the two most abundant shrub species (mountain maple and hazelnut) were selected, one in each of the two following size classes (small ≤ 1 m and large > 1 m). The less frequent shrub species (hobblebush, wild raisin, and honeysuckle) were also sampled using two size classes when encountered in the same sites. In total, 228 individuals were sampled for individual‐based trait measures: 51 mountain maples, 51 hazelnuts, 49 hobblebushes, 38 honeysuckles, and 39 wild raisins.

**TABLE 1:** Sixteen traits chosen for characterization of shrub strategies.

Trait	Abbreviation	Definition
Leaf mass fraction	LMF	Leaf mass as a percent of whole plant mass
Root mass fraction	RMF	Root mass as a percent of whole plant mass
Branch mass fraction	BMF	Branch mass as a percent of whole plant mass
Branch and stem mass fraction	BSMF	Stem and Branch mass as a percent of whole plant mass
Stem mass fraction	SMF	Stem mass as a percent of whole plant mass
Total leaf area (cm^2^)	TLA	Number of leaves of each individual multiplied by LS
Number of leaves	Number of Leaves	
Crown height by diameter ratio	Crown HD	Crown height, divided by crown diameter
Percent crown	Percent crown	Live crown height divided by the total plant height
Leaf Display Index	LDI	TLA divided by the total branch and stem length
Leaf Area Index	LAI	TLA divided by the crown area
Leaf area ratio	LAR	TLA divided by the total plant mass
Lateral extension (cm)	Lateral extension	Branch extension from the root collar to the crown edge
Specific leaf area (g)	SLA	LS divided by the leaf dry mass
Leaf dry matter content	LDMC	Leaf dry mass divided by the fresh mass
Leaf size (cm^2^)	LS	Leaf area

Field sampling occurred in August when most of the summer's growth had already taken place. Vegetation sampling included the height of the shrub (perpendicular to the soil), stem length (height to the first live branch), crown diameter (two measures, the longest and one perpendicular), number of stems per individual (resprouting), and last year's growth using growth scars on the uppermost branch. We counted the number of leaves per shrub, and the total length of branches. We measured lateral extension as the horizontal length from the root collar to the farthest branch length horizontally (Weiher et al., 1999).

Destructive samples of different aerial and underground shrub compartments (leaves, stems, branches, and roots) were collected in August, after full elongation of the leader and full flush of the leaves to control for seasonal variation in traits. For biomass allocation traits all individuals were completely and carefully removed by hand using gardening tools. To measure fresh and dry leaf mass, as well as leaf area, we collected one leaf from each sampled individual. Selected leaves were fully expanded, undamaged, and collected toward the top of the crown (Cornelissen et al., 2003).

### 2.4 Data preparation

Collected branches, stems and roots were dried at 70°C for 3 days, while leaves were dried at 70°C for 1 day (Delagrange et al., 2004). Crown H/D was calculated as the crown height, divided by the crown diameter, we used two measurements to estimate crown diameter; the longest axis and the one perpendicular. Percent Crown was the live crown height divided by the total plant height. Total leaf area (TLA) was calculated using the number of leaves from each individual multiplied by leaf size (LS). LDI was calculated as the TLA divided by the total branch and stem length (cm), LAI was the TLA divided by the crown area (area = *πr* ^2^, using the average of two measurements of crown diameter divided by two) and LAR was expressed as the TLA divided by the total plant mass (Beaudet & Messier, 1998; Delagrange et al., 2004). LS was calculated by scanning one leaf per individual (cm^2^), while SLA was calculated using the LS divided by the leaf dry mass (g) and leaf dry matter content (LDMC) was the dry mass of a leaf divided by its fresh mass (g) (Cornelissen et al., 2003).

### 2.5 Statistical analysis

We compared the abundance of five shrub species considering two gap‐forest environments (gap and forest understory) using a Poisson mixed regression with the random factor as forest understory and gap size classes nested within the landscape identifier (lme4 and AICcmodavg packages). We used a 2 factor ANOVA mixed model to test the effects of species, gap‐forest environment, and interactions on height growth, with the landscape identifier as the random factor (nlme package). The data for height growth were log (*x* + 1) transformed to ensure that data were normal and homoscedastic. Post‐hoc Tukey tests were used to identify categories that differed when an effect was significant.

We tested differences in intraspecific variance using multivariate homogeneity of group variances and Euclidian distance on standardized values of the 16 retained traits (betadisper function in the vegan package). Effects of species interactions with size class and gap‐forest environment were also tested. Overall mean trait values between species, size, and gap‐forest environment groups were tested with multivariate PERMANOVA using Euclidian distance on standardized trait values of 16 traits, allowing full factorial interactions with the landscape identifier as the random factor (adonis function, vegan package). A PERMANOVA test was run a second time with the factor shrub species reduced from five species categories to two contrasting categories: abundant (hazelnut and mountain maple) and less abundant (hobblebush, wild raisin, and honeysuckle) species.

Instead of testing and comparing the main effects on each trait one by one, we generated composite variables using Principal component analysis (PCA). PCA was conducted with normalized values of the 16 traits (ade4, vegan, gclus, and ape packages); height growth and resprouting traits were excluded from these analyses because of the weak contribution of these two traits to the total variance (Borcard et al., 2011). Axis significance was tested using the Kaiser–Guttman and the Broken stick criteria and the variables retained for interpretation of the ordination were the ones with a vector length greater than the radius of the circle of equilibrium (BiodiversityR package) (Borcard et al., 2011).

Significant axes were interpreted as a composite of all significant variables, and the response of these composite variables was stratified by three main effects (species, size class, and gap‐forest environment). Composite variables (using the scores of PCA axes) were used in ANOVAs to test the effects of species, size class, and gap‐forest environment, allowing full factorial interactions, and with the landscape identifier as the random factor (nlme package). Tukey multiple comparisons were used to elucidate differences between levels for the three factors and all interactions. Subsequent three‐factor ANOVAs using the composite variables were run with the factor shrub species reduced from five species categories to two contrasting categories: abundant (hazelnut and mountain maple) and less abundant (hobblebush, wild raisin, and honeysuckle) shrub species. An *α* = .05 was used for all the tests. All analyses were completed using R software (version 3.5.0 – “Joy in Playing”) (R Development Core Team, 2014).

## 3 RESULTS

**FIGURE 1:**
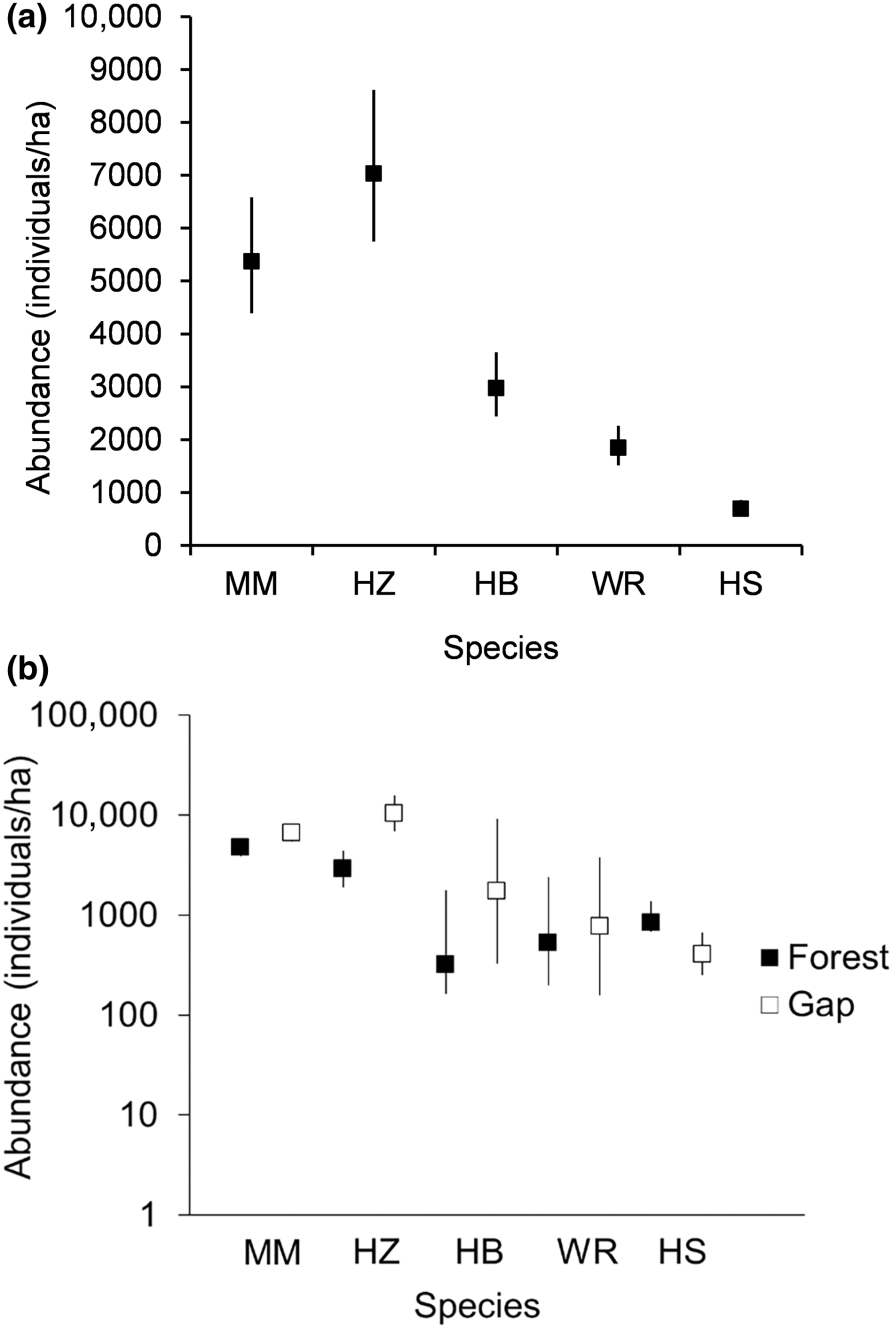
Overall abundance (a) and predicted abundance by gap‐forest environment (b) of five shrubs: (mountain maple [MM], hazelnut [HZ], hobblebush [HB], wild raisin [WR], honeysuckle [HS]. Poisson mixed regression with predicted values and confidence intervals [95%], abundance is in logarithmic scale for b).

**FIGURE 2:**
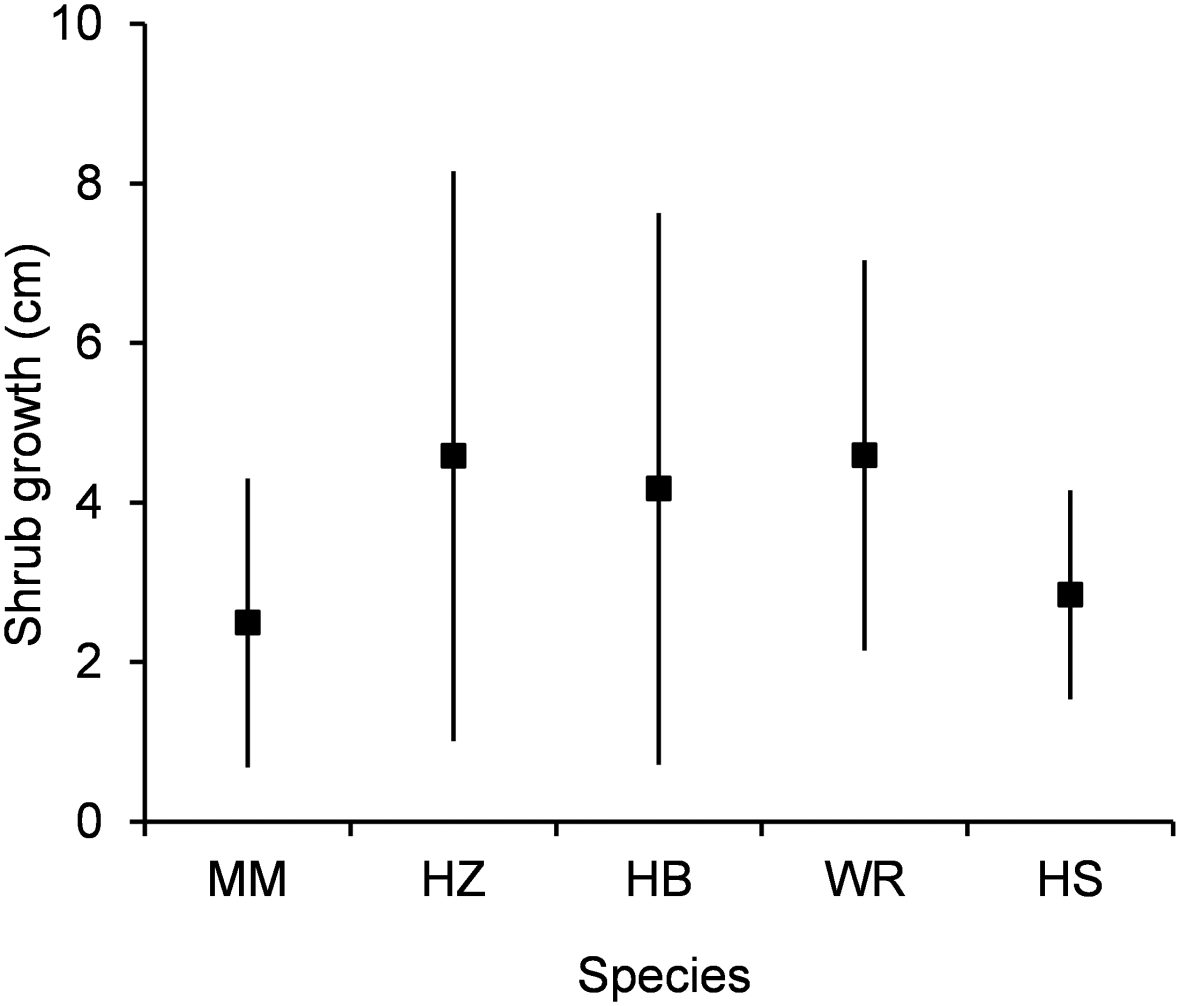
Yearly height growth of five shrubs: (mountain maple [MM], hazelnut [HZ], hobblebush [HB], wild raisin [WR], honeysuckle [HS], means with standard deviations).

Overall, hazelnut (7037 individuals ha^−1^) and mountain maple (5374 individuals ha^−1^) were the most abundant shrubs, followed by hobblebush (2981 individuals ha^−1^), wild raisin (1848 individuals ha^−1^) and finally honeysuckle (699 individuals ha^−1^) (Figure 1a). The differences in densities of all possible one‐on‐one comparisons among five shrub species were significant (*p*(*z*) = <.001) (Figure 1a). Three of the five species displayed significant differences in densities between gap and forest environments (hazelnut: *p*(*z*) = <.001 mountain maple: *p*(*z*) = .004, honeysuckle: *p*(*z*) = .042) (Figure 1b). Hazelnut and mountain maple were both more abundant in gaps, while honeysuckle was the only species more abundant in the forest understory (Figure 1b). Diffuse and direct light levels were 29% and 25%, respectively, in the center of gap openings, and 11% and 12% in forest understory sites. Height growth was lower for mountain maple than for hazelnut (*F* value = 4.53, *p*(*f*) = .001), hobblebush (*F* value = 2.97, *p*(*f*) = .024) and wild raisin (*F* value = 4.53, *p*(*f*) = .001), while wild raisin growth was also greater than that of honeysuckle (*F* value = 2.82, *p*(*f*) = .038) (Figure 2). Although height growth was significantly greater in gaps (Table 2), abundant shrubs (MM, HZ, HB) did not have faster height growth than less abundant ones (WR, HS) (Table 3, Figure 2).

**TABLE 2:** Means and standard deviations of 18 traits across three factors (species: mountain maple [MM], hazelnut [HZ], hobblebush [HB], wild raisin [WR], honeysuckle [HS], size class: large and small, and gap‐forest environment: forest and gap).

	Traits	HB	HS	HZ	MM	WR	Small	Large	Gap	Forest
Crown traits	Crown HD	0.66 ± 0.35	0.91 ± 0.36	0.98 ± 0.33	0.82 ± 0.34	1.21 ± 0.57	0.83 ± 0.41	0.99 ± 0.43	1.00 ± 0.99	0.84 ± 0.83
	Percent crown	0.45 ± 0.21	0.62 ± 0.15	0.61 ± 0.17	0.51 ± 0.21	0.56 ± 0.15	0.54 ± 0.21	0.55 ± 0.17	0.56 ± 0.56	0.53 ± 0.53
Plant morphology	TLA	4078.4 ± 4797	2264.5 ± 2510.6	4047.3 ± 3852.7	2932.9 ± 2893.1	1637.3 ± 1649.7	1091.1 ± 943.5	5402.1 ± 3957.0	3458.3 ± 3458.3	2840.8 ± 2840.8
	LAI	0.73 ± 0.60	0.95 ± 0.50	1.02 ± 0.43	0.92 ± 0.57	1.02 ± 0.65	0.92 ± 0.59	0.93 ± 0.53	0.95 ± 0.94	0.91 ± 0.9
	LDI	10.93 ± 6.15	6.86 ± 3.37	11.86 ± 5.33	13.72 ± 7.59	8.09 ± 3.77	9.24 ± 5.43	12.16 ± 6.47	10.97 ± 10.97	10.34 ± 10.33
	LAR	38.17 ± 20.61	61.97 ± 39.74	39.72 ± 31.14	42.56 ± 58.91	41.16 ± 37.35	51.54 ± 46.80	35.27 ± 29.12	39.40 ± 39.40	47.19 ± 47.18
	Number leaves	37.82 ± 39.49	123.56 ± 114.2	87.36 ± 72.44	42.57 ± 32.59	54.75 ± 47.84	31.83 ± 38.99	107.80 ± 79.84	69.13 ± 69.13	65.76 ± 65.75
	Lateral Extension	76.66 ± 44.76	52.40 ± 25.87	57.83 ± 30.64	70.46 ± 37.63	49.88 ± 34.62	39.92 ± 18.49	88.34 ± 35.73	64.11 ± 64.11	61.26 ± 61.25
Plant allocation	BMF	0.23 ± 0.14	0.25 ± 0.11	0.09 ± 0.07	0.10 ± 0.07	0.18 ± 0.13	0.18 ± 0.14	0.15 ± 0.10	0.16 ± 0.15	0.17 ± 0.17
	LMF	0.09 ± 0.04	0.12 ± 0.05	0.08 ± 0.05	0.07 ± 0.04	0.13 ± 0.1	0.11 ± 0.07	0.08 ± 0.04	0.09 ± 0.09	0.09 ± 0.09
	SMF	0.37 ± 0.15	0.23 ± 0.13	0.21 ± 0.13	0.28 ± 0.16	0.28 ± 0.17	0.24 ± 0.17	0.31 ± 0.14	0.30 ± 0.29	0.26 ± 0.25
	RMF	0.32 ± 0.14	0.42 ± 0.15	0.65 ± 0.2	0.57 ± 0.19	0.42 ± 0.22	0.49 ± 0.24	0.48 ± 0.19	0.47 ± 0.46	0.49 ± 0.49
	BSMF	0.60 ± 0.14	0.47 ± 0.15	0.29 ± 0.16	0.38 ± 0.17	0.46 ± 0.17	0.42 ± 0.2	0.46 ± 0.18	0.45 ± 0.45	0.43 ± 0.42

	Traits	HB	HS	HZ	MM	WR	Small	Large	Gap	Forest
Leaf traits	LS	100.22 ± 34.71	17.90 ± 7.17	43.57 ± 13.18	62.46 ± 25.56	28.79 ± 10.88	46.93 ± 32.76	60.35 ± 38.24	54.85 ± 54.85	51.98 ± 51.98
	SLA	0.029 ± 0.008	0.034 ± 0.010	0.031 ± 0.006	0.032 ± 0.010	0.025 ± 0.005	0.032 ± 0.010	0.028 ± 0.006	0.027 ± 0.008	0.033 ± 0.008
	LDMC	0.34 ± 0.23	0.40 ± 0.18	0.45 ± 0.27	0.32 ± 0.22	0.28 ± 0.13	0.35 ± 0.24	0.37 ± 0.20	0.46 ± 0.46	0.29 ± 0.28
Performance traits	Growth	4.17 ± 3.46	2.84 ± 1.31	4.58 ± 3.57	2.49 ± 1.81	4.59 ± 2.45	3.64 ± 3.06	3.85 ± 2.61	3.88 ± 2.32	3.63 ± 3.18
	Resprouting	2.06 ± 1.20	2.16 ± 1.70	2.02 ± 1.24	2.55 ± 1.97	2.82 ± 1.48	1.93 ± 1.15	2.75 ± 1.84	2.31 ± 1.69	2.31 ± 1.36

**TABLE 3:** ANOVA test table comparing height growth by species (S), size class (SC), and gap‐forest environment (GF).

	*F* value	*p*(*f*)
Species (S)	7.82	<.001
Gap‐forest environment (GF)	5.67	.018
Size class (SC)	3.26	.072
S × GF	0.60	.663
S × SC	0.52	.723
GF × SC	7.69	.006
S × GF × SC	1.42	.229

### 3.1 Shrub traits

The overall means of functional traits significantly differed for each of the three main effects (species, size class, and gap‐forest environment), as well as the two‐way interaction between species and size class (Table 4). PERMANOVA analysis with species groups (abundant and less abundant) also identified significant differences by species group (*F* value = 18.09, *p*(*f*) = .001), size class (*F* value = 24.44, *p*(*f*) = .001) and gap‐forest environment (*F* value = 6.13, *p*(*f*) = .001).

**TABLE 4:** Overall multivariate variation and PERMANOVA overall mean test of 16 shrubs traits across three factors (species [S], size class [SC] and gap‐forest environment [LE]).

	Variability		Mean	
	*F* value	*p*(*f*)	*F* value	*p*(*f*)
Species (S)	0.32	.864	14.80	<.001
Gap‐forest environment (GF)	1.80	.182	7.14	<.001
Size class (SC)	0.33	.564	28.21	<.001
S × GF	1.30	.275	1.41	.061
S × SC	1.48	.214	2.24	.001
GF × SC	NA	NA	1.62	.115
S × GF × SC	NA	NA	1.46	.039

**FIGURE 3:**
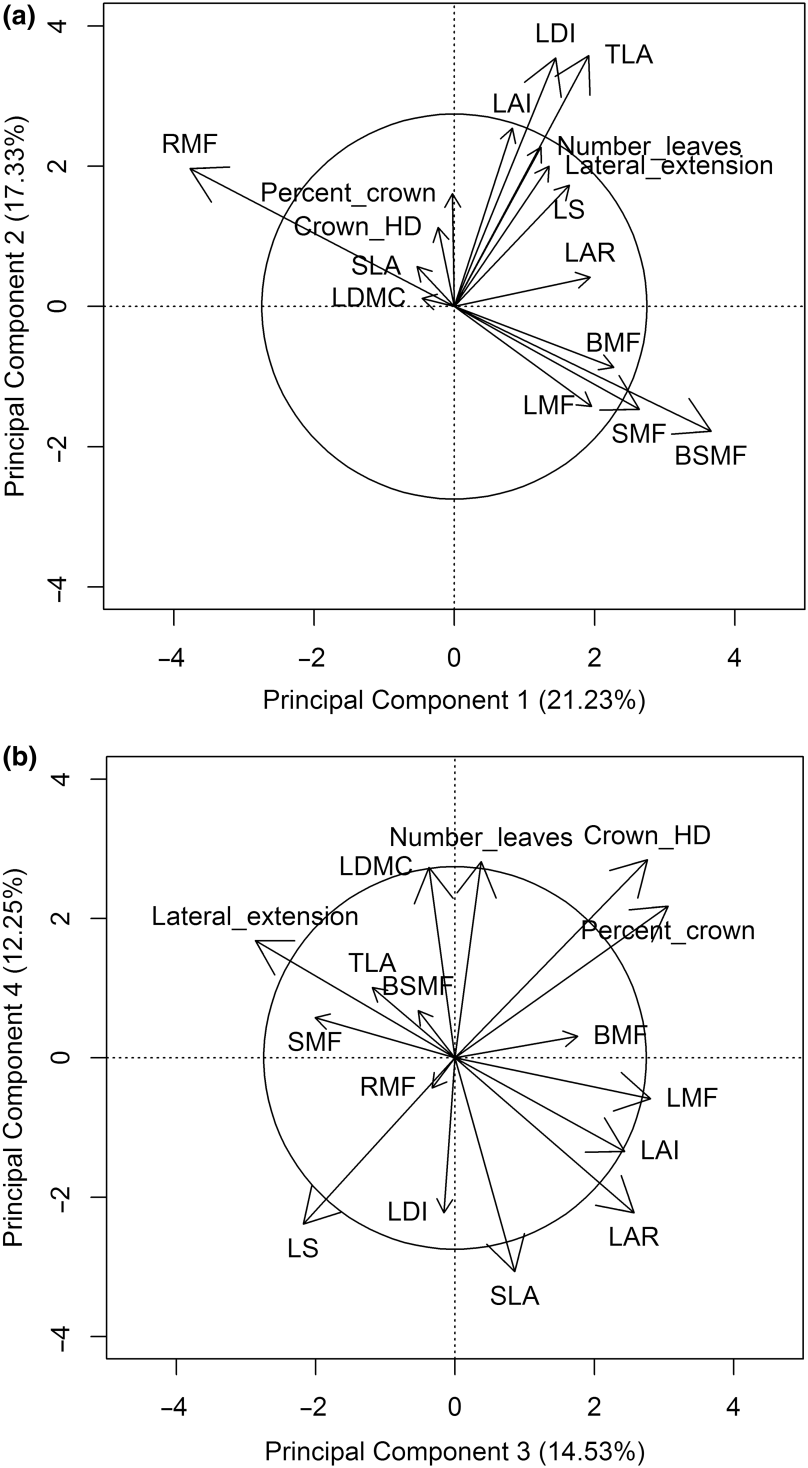
PCA ordination (scaling 2) showing the relationships between 16 field‐measured functional traits from five shrub species, two size classes, and two gap‐forest environments (forest and gaps) with the first four significant dimensions (a: components 1 and 2, and b: components 3 and 4). Significant traits are vectors with greater length than the radius of the circle of equilibrium.

The four primary dimensions of the PCA accounted for 65.34% of the total variance and were all significant according to the Kaiser–Guttman and the broken stick criteria, explaining respectively 21.23%, 17.33%, 14.53%, and 12.25% of the total variance (Figure 3). The first axis (I) was mostly related to and hereafter known as the biomass allocation axis (RMF *r* = −.895 and BSMF *r* = .869), the second (II) correlated to and hereafter known as the leaf display axis (TLA *r* = .767 and LDI *r* = .760), the third (III) hereafter known as crown morphology (Percent Crown *r* = .601, Lateral Extension *r* = −.563) and the fourth (IV) as leaf traits (SLA *r* = −.553) (Figure 3).

Species and size class were significant across all four trait dimensions, while the gap‐forest environment was only different along the leaf trait axis (IV) (Table 5). Furthermore, a significant species group effect (abundant and less abundant) was observed for the biomass allocation axis (I) (*F* value = 41.74, *p*(*f*) = .001), the leaf display axis (II) (*F* value = 49.47, *p*(*f*) = .001) and the leaf traits axis (IV) (*F* value = 4.12, *p*(*f*) = .044).

**TABLE 5:** ANOVA of four themes of trait variation of the PCA (species [S], size class [SC] and gap‐forest environment [GF]).

	Biomass allocation		Leaf display		Crown morphology		Leaf traits	
	Axis 1		Axis 2		Axis 3		Axis 4	
	*F* value	*p*(*f*)	*F* value	*p*(*f*)	*F* value	*p*(*f*)	*F* value	*p*(*f*)
Species (S)	18.92	<.001	14.05	<.001	34.20	<.001	12.43	<.001
Gap‐forest environment (GF)	1.47	.227	0.30	.583	0.21	.648	41.93	<.001
Size class (SC)	16.03	.001	72.04	<.001	42.71	<.001	63.43	<.001
S × GF	2.73	.030	1.19	.316	1.02	.399	0.81	.520
S × SC	3.35	.011	0.80	.524	1.57	.184	3.09	.017
GF × SC	3.46	.064	1.06	.303	1.55	.214	0.17	.684
S × GF × SC	0.96	.433	2.97	.021	0.32	.867	2.17	.074

**FIGURE 4:**
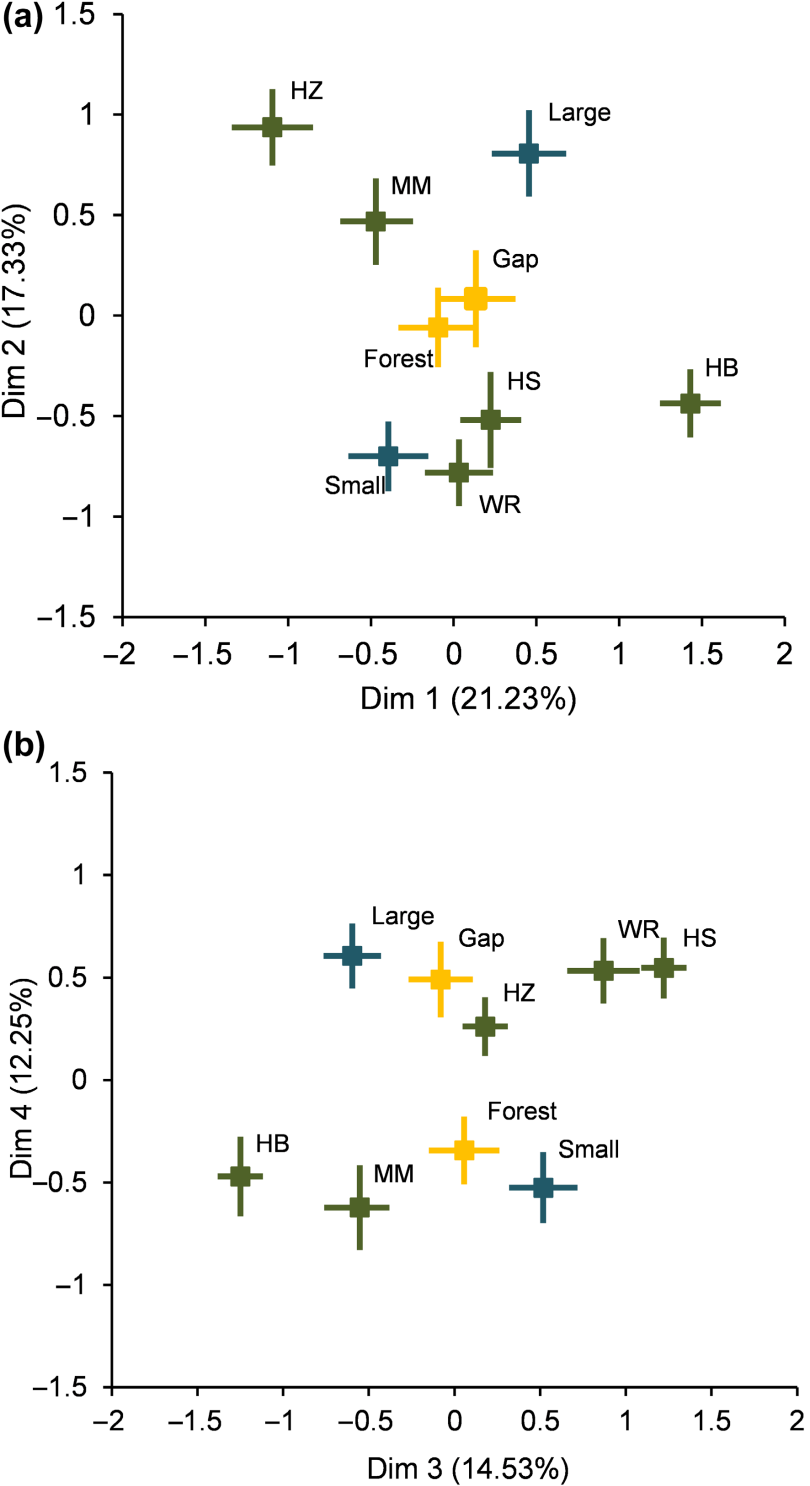
Mean and 95% confidence intervals of the scores of components 1 and 2 (a) and 3 and 4 (b) for each level of the three main effects (species: mountain maple [MM], hazelnut [HZ], hobblebush [HB], wild raisin [WR], honeysuckle [HS], gap‐forest environment: forest and gap, and size class: large and small), of the PCA ordination of 16 field‐measured functional traits (scaling 1).

On the biomass allocation axis I abundant shrubs hazelnut and mountain maple had high RMF and low BSMF values, while the inverse was observed for hobblebush (Figures 3a and 4a). Indeed, on that axis, hobblebush significantly differed from all other species, while hazelnut was different from honeysuckle and wild raisin (Table 6). Mountain maple and hazelnut were associated with higher leaf display values (axis II) (TLA, LDI), while honeysuckle, wild raisin, and hobblebush had lower values (Figures 3a and 4a). Indeed, for leaf display traits (axis II), hobblebush, honeysuckle, and wild raisin were all significantly different from hazelnut and mountain maple (Table 7). On the crown morphology axis III, honeysuckle, and wild raisin were associated with higher Percent Crown and Crown H/D values and lower Lateral Extension values (Figures 3b and 4b). Almost all shrub species were different from each other on the crown morphology principal components axis; the only two species that did not differ significantly were honeysuckle and wild raisin, the two least abundant species (Table 8). Mountain maple and hobblebush were associated with higher SLA (Figures 3b and 4b), with mountain maple and hobblebush being different from all other species except each other on that principal component (axis IV) (Table 9).

**TABLE 6:** Tukey multiple comparisons of main and interaction effects on the biomass allocation axis (I) of the PCA ordination of 16 field‐measured functional traits (scaling 1).

Species	*Z* value	*p*(*z*)
MM ≠ HB	5.91	<.001
HZ ≠ HB	7.87	<.001
HZ ≠ HS	4.17	<.001
HZ ≠ WR	3.29	.009
HS ≠ HB	3.12	.0153
WR ≠ HB	4.08	<.001
Species × Gap‐forest environment		
HZ‐Gap ≠ HZ‐Forest	3.20	.043
Species × Size class		
HZ‐Large ≠ HZ‐Small	4.44	<.010
Gap‐forest environment × Size class		
Small‐Gap ≠ Large‐Gap	3.47	.003

*Note*: Only significant differences are presented (species: mountain maple [MM], hazelnut [HZ], hobblebush [HB], wild raisin [WR], honeysuckle [HS], gap‐forest environment: forest and gap, and size class: large and small).

**TABLE 7:** Tukey multiple comparisons of main and interaction effects on the leaf display axis (II) of the PCA ordination of 16 field‐measured functional traits (scaling 1).

Species	*Z* value	*p*(*z*)
MM ≠ HB	3.56	.003
MM ≠ HS	2.85	.035
MM ≠ WR	4.42	<.001
HZ ≠ HB	5.29	<.001
HZ ≠ HS	4.46	<.001
HZ ≠ WR	6.05	<.001

*Note*: Only significant differences are presented (species: mountain maple [MM], hazelnut [HZ], hobblebush [HB], wild raisin [WR], honeysuckle [HS], gap‐forest environment: forest and gap, and size class: large and small).

**TABLE 8:** Tukey multiple comparisons of main and interaction effects on plant morphology axis (III) of the PCA ordination of 16 field‐measured functional traits (scaling).

Species	*Z* value	*p*(*z*)
MM ≠ HB	2.86	.034
MM ≠ HS	6.77	<.001
MM ≠ HZ	3.22	.011
HZ ≠ HB	6.04	<.001
HZ ≠ HS	3.81	.001
HS ≠ HB	9.34	<.001
WR ≠ HB	8.50	<.001
WR ≠ HZ	2.89	.032
WR ≠ MM	5.88	<.001

*Note*: Only significant differences are presented (species: mountain maple [MM], hazelnut [HZ], hobblebush [HB], wild raisin [WR], honeysuckle [HS], gap‐forest environment: forest and gap, and size class: large and small).

**TABLE 9:** Tukey multiple comparisons of main and interaction effects on the leaf traits axis (IV) of the PCA ordination of 16 field‐measured functional traits (scaling 1).

Species	*Z* value	*p*(*z*)
MM ≠ HS	5.73	<.001
MM ≠ HZ	4.10	<.001
HZ ≠ HB	3.56	.003
HS ≠ HB	5.21	<.001
WR ≠ HB	4.50	<.001
WR ≠ MM	5.01	<.001
Species × Size class		
HS‐Small ≠ HS‐Large	5.52	<.010
HZ‐Small ≠ HZ‐Large	3.51	.016

*Note*: Only significant differences are presented (species: mountain maple [MM], hazelnut [HZ], hobblebush [HB], wild raisin [WR], and honeysuckle [HS]).

### 3.2 Ontogeny

**FIGURE 5:**
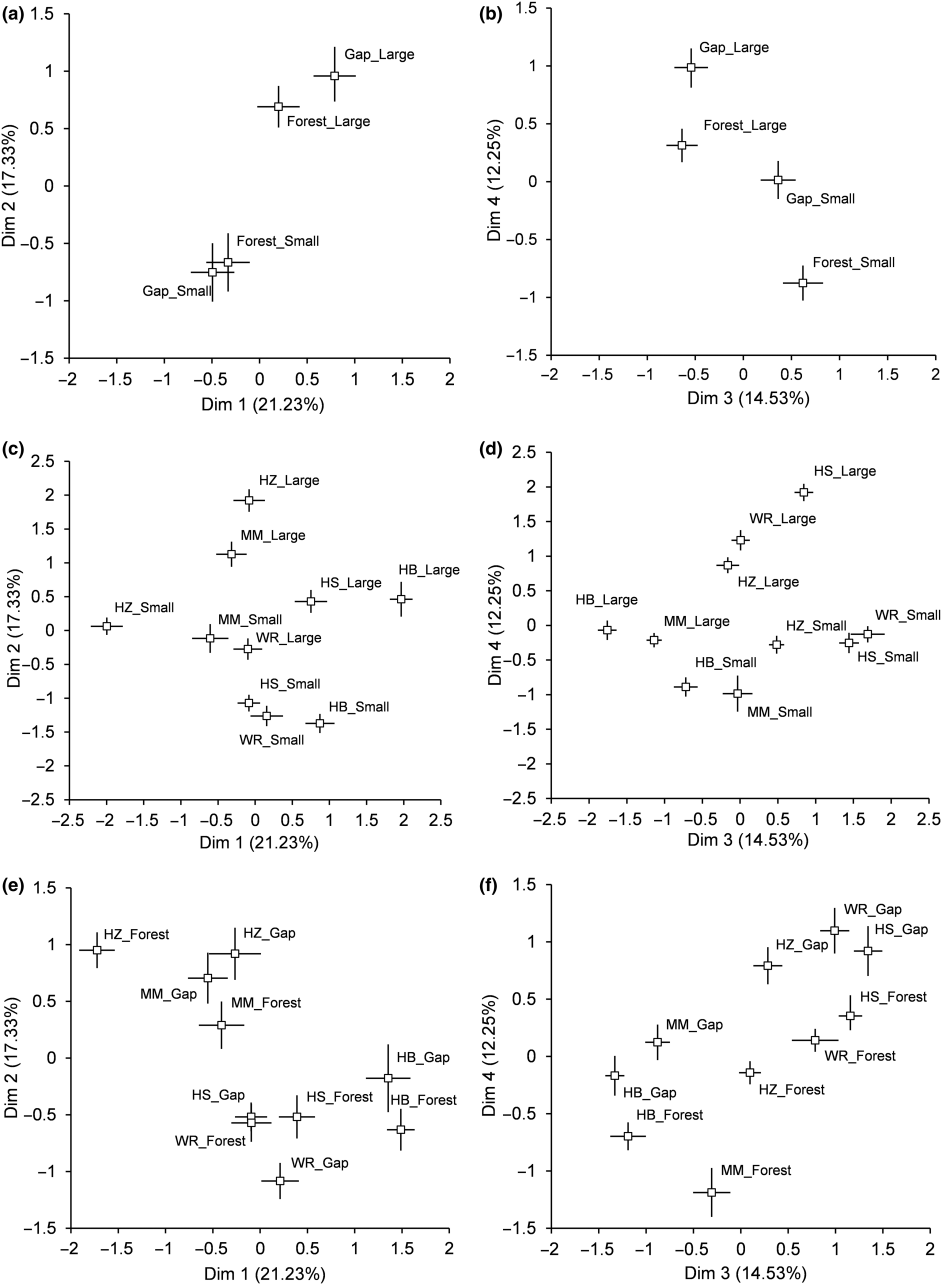
Mean and 95% confidence intervals of the scores for each level of the Size Class × Light interaction effect of components 1 and 2 (a) and 3 and 4 (b), of the Species × Size Class interaction effect of components 1 and 2 (c) and 3 and 4 (d), and Species × Light interaction effect of components 1 and 2 (e) and 3 and 4 (f) (species: mountain maple [MM], hazelnut [HZ], hobblebush [HB], wild raisin [WR], honeysuckle [HS], gap‐forest environment: forest and gap, and size class: large and small), of the PCA ordination of 16 field‐measured functional traits (scaling 1).

In general, large individuals invested significantly more in BSMF than small individuals (axis I) and had greater leaf display (axis II) (high value of TLA and LDI) (Figures 3a and 4a). Larger individuals also had greater Lateral Extension, smaller Crown H/D ratio, smaller Percent Crown (axis III), and lower SLA (axis IV) (Figures 3b and 4b). Size class differences were most accentuated for hazelnuts as expressed by the significant species and size class interaction on the biomass allocation axis (I) (Table 6) and the leaf traits axis (IV) (Table 9). For hazelnut, small individuals tended to have greater RMF, and greater SLA (Figure 5c,d).

### 3.3 Gap‐forest growth environment

We detected very little effect of the growing environment (gap or forest) on traits associated with biomass allocation (axis I), leaf display (axis II), or crown morphology (axis III). However, leaf traits (axis IV) significantly changed according to the gap‐forest environment. Greater SLA was observed within the understory forest environment (Figures 3b and 4b). The significant interaction between hazelnut and gap‐forest environment on the biomass allocation axis suggested that hazelnut increased allocation to root biomass in the forested environments (Table 6, Figure 5e,f).

### 3.4 Intraspecific variance

Overall trait intraspecific variance did not differ between species, size classes, or gap‐forest environments for the 16 measured traits (Table 4).

## 4 DISCUSSION

### 4.1 Gap specialists and growth

We hypothesized that abundant shrubs would be gap specialists (Royo & Carson, 2006; Young & Peffer, 2010). Although abundant shrubs have higher densities in gaps, they also have far greater densities than the other shrubs in forest understories. In contrast, the abundance of the three less abundant shrub species was equivalent between the two environments or more abundant in the forest, thus lending partial support to this hypothesis. The greater abundance of beaked hazel and mountain maple in gaps and forest understories suggests that these species were able to respond to changes in environmental conditions.

To further explore these hypotheses, we also evaluated growth between the two gap‐forest environments. We expected that abundant shrubs would display fast growth (Young & Peffer, 2010). Generally, abundant shrubs hazelnut and mountain maple did not grow faster than less abundant shrubs. In fact, the second most abundant shrub, mountain maple, had lower growth than most other shrubs. We thus find weak support for our first hypothesis that the most abundant shrubs use faster growth to be gap specialists.

### 4.2 Persistence and carbon allocation

Our hypotheses led us to question what traits allowed abundant shrubs to be successful. We found that abundant shrubs had higher RMF, TLA, LDI, and lower Percent Crown and Crown H/D. Indeed, functional traits influence aspects of demographic rates such as abundance, immigration, growth, reproduction, and survival (Adler et al., 2014; HilleRisLambers et al., 2012; Shipley et al., 2016; van der Sande et al., 2018).

Hazelnut and mountain maple were the two most abundant shrubs, and they both had high RMF (65% and 57% respectively). These RMF values are at the upper end of values reported in the literature where the RMF of woody shrubs and small trees averages 32%, but varied from 16% to 63% (Poorter et al., 2012).

Our results suggest that abundant shrubs invest more in RMF to exploit what has been termed the persistence niche (Bond & Midgley, 2001; Markesteijn & Poorter, 2009; Ottaviani et al., 2020). Ubiquitous low investment to stem support structures among shrubs appears to identify a strategy that may be unique to understory species. In our study, abundant shrubs in temperate southeastern mixedwood forests invest in root biomass, extensive and efficient leaf display, and a monolayer crown to persist in forests and deploy a “sit and wait” strategy to rapidly colonize new areas following canopy disturbance (Fraterrigo, 2016). Indeed, underground structures may provide increased survival during difficult periods, and allow fast recovery after disturbance (Klimešová et al., 2018; Ottaviani et al., 2020).

RMF correlates positively with resource conservation (i.e., reserve storage) and rooting depth for soil exploration in situations of nutrient stress, whereas greater SRL (specific root length) may correspond to resource acquisition (Freschet et al., 2018; Larson & Funk, 2016). Coarse roots support above‐ground structures, and function as storage organs as their absorptive ability is almost completely lost (Cheng et al., 2005; Klimešová et al., 2018). Mountain maple reproduced by basal sprouts and stem layering, while hazelnut used specialized underground stems for reproduction (Jobidon, 1995).

Tree species display an acrotonic development leading to the accumulation of large architectural structures (Barthélémy & Caraglio, 2007). This can be an important carbon balance constraint (Delagrange et al., 2004), especially when passing through the recruitment phase, from shade to the top of the canopy. In contrast, shrub species exhibit a basitonic development which means they invest more carbon in basal parts, staying small and displaying laterally (Barthélémy & Caraglio, 2007). In addition to avoiding large maintenance costs, basitonic development also explains why shrubs can rapidly reshape their morphology according to resource availability through height reduction and branch shedding (Aubin et al., 2005).

### 4.3 Other shrub trait strategies

Mountain maple and hazelnut were positively associated with the leaf display axis (II) (LDI and TLA). These two shrubs maintained high leaf display (TLA), while simultaneously sustaining high LDI, implying that the two most abundant shrubs increased their leaf area in an efficient manner in low light, by using less branch and stem length per unit leaf area. The intermediately abundant hobblebush was strongly associated with aboveground biomass (BSMF). Hobblebush had the highest TLA of the five species, suggesting a remarkable ability to display a large leaf area while having the lowest within crown shading (LAI) of the five species (Table 2). Hobblebush and mountain maple were negatively associated with the crown morphology axis (III), thus maintaining the lowest values for Percent Crown and Crown H/D of the five species (Table 2), and therefore displaying archetype shade tolerant crown features including a wide crown width and a shallow crown depth (Messier et al., 1999; Niinemets, 2010).

Honeysuckle and wild raisin, the two least abundant shrubs, had higher values for Percent Crown, entailing small crown area and large crown depth, characteristic of shade intolerants (Claveau et al., 2002; Delagrange et al., 2004; Messier et al., 1999).

Variation in leaf traits was characterized by a negative relationship between SLA and LDMC and lower SLA in high light and large individuals, similar to the established literature (Niinemets, 2006, 2010; Poorter, 1999, 2002). We expected high SLA to be positively related to growth and competitive ability (Weiher et al., 1999), but no relationship was found in our study. SLA is potentially a poor indicator of trait strategy due to its plasticity (Adler et al., 2014). The relatively low light levels within the small gap dynamics of the southern mixedwood forests in this study likely contributed to the importance of morphological traits, while in higher light conditions physiological traits would have become more important (Delagrange et al., 2004; Poorter, 1999).

### 4.4 Ontogeny

For the same species, small individuals had higher RMF values while large individuals had greater BSMF values on the biomass allocation axis (I). Shifts in traits with ontogeny are also observed for trees where plant size is positively correlated with SMF, and negatively correlated with RMF (Delagrange et al., 2004; Poorter, 1999; Poorter et al., 2012). Although this ontogeny trend common in trees is intuitive in species that can attain the canopy, in shrubs it may reflect a transfer to capturing light as growth environments change.

Larger individuals were negatively correlated with crown morphology traits (Percent Crown and Lateral Extension), suggesting that shrubs develop an increasing monolayer canopy with increasing size. Previous work proposes that small shade‐tolerant individuals develop a monolayered canopy, while larger individuals gradually develop a multilayered canopy as they are exposed to increasing sunlight (Delagrange et al., 2004; Niinemets, 2010). Decreasing light interception efficiency with increasing size suggests that smaller individuals are more shade tolerant (Delagrange et al., 2006; Kneeshaw et al., 2006). It is probable that large shrubs develop a monolayer canopy with larger sizes because they have little chance of being released from the shade and must retain shade‐adapted canopy morphology throughout ontogeny.

### 4.5 Intraspecific variance

We postulated that abundant shrubs would be more plastic to take advantage of fast growth in high light and biomass shutdown in low light (Pugnaire & Valladares, 2007). Differences in intraspecific variance were not detected. As shrubs need to combine persistence and colonization abilities at the same time, trait compromises observed between tree species must occur within the same shrub species, which would require greater trait plasticity.

## 5 CONCLUSION

We sought to answer the fundamental question; what makes a particular shrub species successful or more abundant in a forested landscape driven by intermediate natural disturbances? Contrary to expectations, shrub growth, and trait intraspecific variance did not contribute much to explain the difference between more abundant and less abundant species. All species exhibited similar levels of variation in traits, whatever the environment. Rather, we found that abundant shrub species display a suite of traits that are adapted to persist in the forest understory by investing in root biomass and monolayer crowns, as opposed to less abundant shrubs. Future work could include consideration of additional shrub traits, especially with regard to the colonization processes (seed production, dispersion, vegetative layering, etc.) that confer shrubs' competitive abilities after forest openings.

These questions are at the heart of not only fundamental, but also practical goals such as the management of competitive shrub and non‐commercial tree species to provide better tree regeneration in our forests. Degraded balsam fir/yellow birch forests are common in this transition zone mostly due to past high‐grading harvests. They have been characterized by a subsequent invasion of the abundant shrub species studied here, causing stand yield stagnation. Our results partially explain the “explosion” of these shrub populations after the high‐grading era. Their traits are associated with persistence and the sit‐and‐wait strategy, such as high RMF, which explains why they have been such strong competitors following small canopy openings. Presently, small‐scale harvesting using irregular shelterwood silviculture is still the most commonly applied treatment in that forest type, indicating that the shrub invasion phenomenon could be still exacerbated by forestry. We highly recommend monitoring shrub populations at the regional scale for a better portrayal of the evolution over the last decades as a response to disturbances.

## AUTHOR CONTRIBUTIONS

**Rudiger Markgraf:** Conceptualization (equal); data curation (lead); formal analysis (lead); investigation (equal); methodology (equal); project administration (equal); writing – original draft (lead); writing – review and editing (equal). **Frédérik Doyon:** Conceptualization (equal); data curation (supporting); formal analysis (equal); funding acquisition (lead); investigation (equal); methodology (equal); project administration (lead); resources (lead); software (lead); supervision (lead); validation (equal); visualization (equal); writing – original draft (supporting); writing – review and editing (equal). **Sylvain Delagrange:** Conceptualization (equal); investigation (equal); methodology (equal); supervision (supporting); validation (supporting); writing – original draft (supporting); writing – review and editing (supporting). **Daniel Kneeshaw:** Conceptualization (equal); funding acquisition (lead); investigation (equal); methodology (equal); project administration (equal); supervision (lead); validation (equal); visualization (equal); writing – original draft (supporting); writing – review and editing (lead).

## Supporting information


Appendix S1

## Data Availability

The data that support the findings of this study will be openly available in the TOPIC network (upon acceptance of this manuscript). Our data and R code for this paper are equally accessible in Supporting Information.

## References

[ece310774-bib-0001] Adler, P. B. , Salguero‐Gómez, R. , Compagnoni, A. , Hsu, J. S. , Ray‐Mukherjee, J. , Mbeau‐Ache, C. , & Franco, M. (2014). Functional traits explain variation in plant life history strategies. Proceedings of the National Academy of Sciences of the United States of America, 111, 740–745. 24379395 10.1073/pnas.1315179111 PMC3896207

[ece310774-bib-0002] Alpert, P. , & Simms, E. L. (2002). The relative advantages of plasticity and fixity in different environments: When is it good for a plant to adjust? Evolutionary Ecology, 16, 285–297.

[ece310774-bib-0003] Aubin, I. , Messier, C. , & Kneeshaw, D. (2005). Population structure and growth acclimation of mountain maple along a successional gradient in the southern boreal forest. Ecoscience, 12, 540–548.

[ece310774-bib-0004] Aubin, I. , Munson, A. D. , Cardou, F. , Burton, P. J. , Isabel, N. , Pedlar, J. H. , Paquette, A. , Taylor, A. R. , Delagrange, S. , Kebli, H. , & Messier, C. (2016). Traits to stay, traits to move: A review of functional traits to assess sensitivity and adaptive capacity of temperate and boreal trees to climate change. Environmental Reviews, 24, 164–186.

[ece310774-bib-0005] Barthélémy, D. , & Caraglio, Y. (2007). Plant architecture: A dynamic, multilevel and comprehensive approach to plant form, structure and ontogeny. Annals of Botany, 99, 375–407. 17218346 10.1093/aob/mcl260 PMC2802949

[ece310774-bib-0006] Beaudet, M. , & Messier, C. (1998). Growth and morphological responses of yellow birch, sugar maple, and beech seedlings growing under a natural light gradient. Canadian Journal of Forest Research, 28, 1007–1015.

[ece310774-bib-0007] Beaudet, M. , & Messier, C. (2002). Variation in canopy openness and light transmission following selection cutting in northern hardwood stands: An assessment based on hemispherical photographs. Agricultural and Forest Meteorology, 110, 217–228.

[ece310774-bib-0008] Bell, F. W. , Kershaw, M. , Aubin, I. , Thiffault, N. , Dacosta, J. , & Wiensczyk, A. (2011). Ecology and traits of plant species that compete with boreal and temperate forest conifers: An overview of available information and its use in forest management in Canada. The Forestry Chronicle, 87, 161–174.

[ece310774-bib-0009] Bond, W. J. , & Midgley, J. J. (2001). Ecology of sprouting in woody plants: The persistence niche. Trends in Ecology and Evolution, 16, 45–51. 11146144 10.1016/s0169-5347(00)02033-4

[ece310774-bib-0010] Bond, W. J. , & Midgley, J. J. (2003). The evolutionary ecology of sprouting in woody plants. International Journal of Plant Sciences, 164, S103–S114.

[ece310774-bib-0011] Borcard, D. , Gillet, F. , & Legendre, P. (2011). Numerical ecology with R. Springer.

[ece310774-bib-0012] Cheng, S. , Widden, P. , & Messier, C. (2005). Light and tree size influence belowground development in yellow birch and sugar maple. Plant and Soil, 270, 321–330.

[ece310774-bib-0013] Claveau, Y. , Messier, C. , Comeau, P. G. , & Coates, K. D. (2002). Growth and crown morphological responses of boreal conifer seedlings and saplings with contrasting shade tolerance to a gradient of light and height. Canadian Journal of Forest Research, 32, 458–468.

[ece310774-bib-0014] Cornelissen, J. H. C. , Lavorel, S. , Garnier, E. , Díaz, S. , Buchmann, N. , Gurvich, D. E. , Reich, P. B. , Ter Steege, H. , Morgan, H. D. , Van Der Heijden, M. G. A. , Pausas, J. G. , & Poorter, H. (2003). A handbook of protocols for standardised and easy measurement of plant functional traits worldwide. Australian Journal of Botany, 51, 335–380.

[ece310774-bib-0015] Delagrange, S. , Messier, C. , Lechowicz, M. J. , & Dizengremel, P. (2004). Physiological, morphological and allocational plasticity in understory deciduous trees: Importance of plant size and light availability. Tree Physiology, 24, 775–784. 15123449 10.1093/treephys/24.7.775

[ece310774-bib-0016] Delagrange, S. , Montpied, P. , Dreyer, E. , Messier, C. , & Sinoquet, H. (2006). Does shade improve light interception efficiency? A comparison among seedlings from shade‐tolerant and ‐intolerant temperate deciduous tree species. New Phytologist, 172, 293–304. 16995917 10.1111/j.1469-8137.2006.01814.x

[ece310774-bib-0017] Doyon, F. , & Lafleur, B. (2004). Caractérisation de la structure et du dynamisme des peuplements mixtes à bouleau jaune: pour une sylviculture irrégulière proche de la nature. Institut Québécois d'Aménagement de la Forêt Feuillue.

[ece310774-bib-0018] Fraterrigo, J. M. (2016). Land‐use legacies and the role of persistence traits in species recovery. Applied Vegetation Science, 19, 555–556.

[ece310774-bib-0019] Freschet, G. T. , Swart, E. M. , & Cornelissen, J. H. (2015). Integrated plant phenotypic responses to contrasting above‐and below‐ground resources: Key roles of specific leaf area and root mass fraction. New Phytologist, 206, 1247–1260. 25783781 10.1111/nph.13352

[ece310774-bib-0020] Freschet, G. T. , Violle, C. , Bourget, M. Y. , Scherer‐Lorenzen, M. , & Fort, F. (2018). Allocation, morphology, physiology, architecture: The multiple facets of plant above‐and below‐ground responses to resource stress. New Phytologist, 219, 1338–1352. 29856482 10.1111/nph.15225

[ece310774-bib-0021] Gaucher, C. , Gougeon, S. , Mauffette, Y. , & Messier, C. (2005). Seasonal variation in biomass and carbohydrate partitioning of understory sugar maple (*Acer saccharum*) and yellow birch (*Betula alleghaniensis*) seedlings. Tree Physiology, 25, 93–100. 15519990 10.1093/treephys/25.1.93

[ece310774-bib-0022] Gleeson, S. K. , & Tilman, D. (1994). Plant allocation, growth rate and succesional status. Functional Ecology, 8, 543–550.

[ece310774-bib-0023] HilleRisLambers, J. , Adler, P. B. , Harpole, W. S. , Levine, J. M. , & Mayfield, M. M. (2012). Rethinking community assembly through the lens of coexistence theory. Annual Review of Ecology, Evolution, and Systematics, 43, 227–248.

[ece310774-bib-0024] Jobidon, R. (1995). Autécologie de quelques espèces de compétition d'importance pour la régénération forestière au Quebec: revue de littérature. Gouvernement du Québec.

[ece310774-bib-0025] Kitajima, K. (1994). Relative importance of photosynthetic traits and allocation patterns as correlates of seedling shade tolerance of 13 tropical trees. Oecologia, 98, 419–428. 28313920 10.1007/BF00324232

[ece310774-bib-0026] Klimešová, J. , Martínková, J. , & Ottaviani, G. (2018). Belowground plant functional ecology: Towards an integrated perspective. Functional Ecology, 32, 2115–2126.

[ece310774-bib-0027] Kneeshaw, D. D. , Kobe, R. K. , Coates, K. D. , & Messier, C. (2006). Sapling size influences shade tolerance ranking among southern boreal tree species. Journal of Ecology, 94, 471–480.

[ece310774-bib-0028] Kneeshaw, D. D. , & Prévost, M. (2007). Natural canopy gap disturbances and their role in maintaining mixed‐species forests of central Quebec, Canada. Canadian Journal of Forest Research, 37, 1534–1544.

[ece310774-bib-0029] Kumordzi, B. B. , Aubin, I. , Cardou, F. , Shipley, B. , Violle, C. , Johnstone, J. , Anand, M. , Arsenault, A. , Bell, F. W. , Bergeron, Y. , & Boulangeat, I. (2019). Geographic scale and disturbance influence intraspecific trait variability in leaves and roots of North American understorey plants. Functional Ecology, 33, 1771–1784.

[ece310774-bib-0030] Larson, J. E. , & Funk, J. L. (2016). Seedling root responses to soil moisture and the identification of a belowground trait spectrum across three growth forms. New Phytologist, 210, 827–838. 26765506 10.1111/nph.13829

[ece310774-bib-0031] Markesteijn, L. , & Poorter, L. (2009). Seedling root morphology and biomass allocation of 62 tropical tree species in relation to drought‐ and shade‐tolerance. Journal of Ecology, 97, 311–325.

[ece310774-bib-0032] McCarthy, M. C. , & Enquist, B. J. (2007). Consistency between an allometric approach and optimal partitioning theory in global patterns of plant biomass allocation. Functional Ecology, 21, 713–720.

[ece310774-bib-0033] Messier, C. , Doucet, R. , Ruel, J. , Claveau, Y. , Kelly, C. , & Lechowicz, M. J. (1999). Functional ecology of advance regeneration in relation to light in boreal forests. Canadian Journal of Forest Research, 29, 812–823.

[ece310774-bib-0034] Niinemets, Ü. (2006). The controversy over traits conferring shade‐tolerance in trees: Ontogenetic changes revisited. Journal of Ecology, 94, 464–470.

[ece310774-bib-0035] Niinemets, Ü. (2010). A review of light interception in plant stands from leaf to canopy in different plant functional types and in species with varying shade tolerance. Ecological Research, 25, 693–714.

[ece310774-bib-0036] Ottaviani, G. , Molina‐Venegas, R. , Charles‐Dominique, T. , Chelli, S. , Campetella, G. , Canullo, R. , & Klimešová, J. (2020). The neglected belowground dimension of plant dominance. Trends in Ecology & Evolution, 35, 763–766. 32650986 10.1016/j.tree.2020.06.006

[ece310774-bib-0037] Poorter, H. (2002). Plant growth and carbon economy. *Encyclopidia of Life Sciences*. 10.1038/npg.els.0003200

[ece310774-bib-0038] Poorter, H. , Niklas, K. J. , Reich, P. B. , Oleksyn, J. , Poot, P. , & Mommer, L. (2012). Biomass allocation to leaves, stems and roots: Meta‐analysis of interspecific variation and environmental control. New Phytologist, 193, 30–50. 22085245 10.1111/j.1469-8137.2011.03952.x

[ece310774-bib-0039] Poorter, L. (1999). Growth responses of 15 rain‐forest tree species to a light gradient: The relative importance of morphological and physiological traits. Functional Ecology, 13, 396–410.

[ece310774-bib-0040] Poorter, L. , & Werger, M. J. A. (1999). Light environment, sapling architecture, and leaf display in six rain forest tree species. American Journal of Botany, 86, 1464–1473. 10523286

[ece310774-bib-0041] Prévost, M. , Roy, V. , & Raymond, P. (2003). Sylviculture et régénération des forêts mixtes du Québec (Canada): une approche qui respecte la dynamique naturelle des peuplements. Gouvernement du Québec.

[ece310774-bib-0042] Pugnaire, F. , & Valladares, F. (2007). Functional plant ecology. Taylor and Francis.

[ece310774-bib-0043] R Development Core Team . (2014). R: A language and environment for statistical computing. R Foundation for Statistical Computing.

[ece310774-bib-0044] Reich, P. B. , Ellsworth, D. S. , Walters, M. B. , Vose, J. M. , Gresham, C. , Volin, J. C. , & Bowman, W. D. (1999). Generality of leaf trait relationships: A test across six biomes. Ecology, 80, 1955–1969.

[ece310774-bib-0045] Reich, P. B. , Wright, I. J. , Cavender‐Bares, J. , Craine, J. M. , Oleksyn, J. , Westoby, M. , & Walters, M. B. (2003). The evolution of plant functional variation: Traits, spectra, and strategies. International Journal of Plant Sciences, 164, S143–S164.

[ece310774-bib-0046] Robitaille, A. , & Saucier, J.‐P. (1998). Paysages régionaux du Québec méridional. Publications du Québec.

[ece310774-bib-0047] Royo, A. A. , & Carson, W. P. (2006). On the formation of dense understory layers in forests worldwide: Consequences and implications for forest dynamics, biodiversity, and succession. Canadian Journal of Forest Research, 36, 1345–1362.

[ece310774-bib-0048] Sabbagh, P. , Nolet, P. , Doyon, F. , & Talbot, J.‐F. (2002). Classification et caractérisation des forêts dégradées de l'Outaouais. Institut Québécois d'Aménagement de la Forêt Feuillue.

[ece310774-bib-0049] Shipley, B. , De Bello, F. , Cornelissen, J. H. C. , Laliberté, E. , Laughlin, D. C. , & Reich, P. B. (2016). Reinforcing loose foundation stones in trait‐based plant ecology. Oecologia, 180, 923–931. 26796410 10.1007/s00442-016-3549-x

[ece310774-bib-0050] Taugourdeau, O. , Delagrange, S. , Lecigne, B. , Sousa‐Silva, R. , & Messier, C. (2019). Sugar maple (*Acer saccharum* Marsh.) shoot architecture reveals coordinated ontogenetic changes between shoot specialization and branching pattern. Trees, 33, 1615–1625.

[ece310774-bib-0052] van der Sande, M. T. , Arets, E. J. , Peña‐Claros, M. , Hoosbeek, M. R. , Cáceres‐Siani, Y. , van der Hout, P. , & Poorter, L. (2018). Soil fertility and species traits, but not diversity, drive productivity and biomass stocks in a Guyanese tropical rainforest. Functional Ecology, 32, 461–474.

[ece310774-bib-0054] Weiher, E. , Van Der Werf, A. , Thompson, K. , Roderick, M. , Garnier, E. , & Eriksson, O. (1999). Challenging Theophrastus: A common core list of plant traits for functional ecology. Journal of Vegetation Science, 10, 609–620.

[ece310774-bib-0055] Westoby, M. , Falster, D. S. , Moles, A. T. , Vesk, P. A. , & Wright, I. J. (2002). Plant ecological strategies: Some leading dimensions of variation between species. Annual Review of Ecology and Systematics, 33, 125–159.

[ece310774-bib-0056] Woods, K. D. (2000). Dynamics in late‐successional hemlock–hardwood forests over three decades. Ecology, 81, 110–126.

[ece310774-bib-0057] Wright, I. J. , Reich, P. B. , Westoby, M. , Ackerly, D. D. , Baruch, Z. , Bongers, F. , Cavender‐Bares, J. , Chapin, T. , Cornelissen, J. H. C. , Diemer, M. , Flexas, J. , Garnier, E. , Groom, P. K. , Gulias, J. , Hikosaka, K. , Lamont, B. B. , Lee, T. , Lee, W. , Lusk, C. , … Villar, R. (2004). The worldwide leaf economics spectrum. Nature, 428, 821–827. 15103368 10.1038/nature02403

[ece310774-bib-0058] Wright, S. J. , Kitajima, K. , Kraft, N. J. B. , Reich, P. B. , Wright, I. J. , Bunker, D. E. , Condit, R. , Dalling, J. W. , Davies, S. J. , Díaz, S. , Engelbrecht, B. M. J. , Harms, K. E. , Hubbell, S. P. , Marks, C. O. , Ruiz‐Jaen, M. C. , Salvador, C. M. , & Zanne, A. E. (2010). Functional traits and the growth‐mortality trade‐off in tropical trees. Ecology, 91, 3664–3674. 21302837 10.1890/09-2335.1

[ece310774-bib-0059] Young, T. P. , & Peffer, E. (2010). “Recalcitrant understory layers” revisited: Arrested succession and the long life‐spans of clonal mid‐successional species. Canadian Journal of Forest Research, 40, 1184–1188.

